# Quality of antenatal care provision in rural villages of Satna district, Madhya Pradesh, India: a quantitative formative study to help the development of an evidence-based contextualized complex health intervention of the CHAMPION2 cluster randomized trial

**DOI:** 10.1186/s12884-026-08925-5

**Published:** 2026-03-23

**Authors:** Siddharudha Shivalli, Ila Fazzio, Diana Elbourne, Sridevi Karnati, Harshavardhan Reddy, Padmanabh Reddy, Rakhi Nair, Madan Gopal, Peter Boone, Chris Frost

**Affiliations:** 1 London School of Hygiene and Tropical Medicine, London, UK; 2 Effective Intervention, London, UK; 3 GH Training and Consulting, Hyderabad, India; 4 NICE Foundation, Hyderabad, India

**Keywords:** Antenatal care, Formative study, India, Quality, Provision of care, Rural, Village

## Abstract

**Background:**

Since 2005, maternal and newborn deaths have declined in India. Nonetheless, if the current mortality trends continue, India may not achieve the Sustainable Development Goal targets without enhancing the quality of care across the continuum from pregnancy to delivery, particularly in poorly performing states. This study aimed to help the development of an evidence-based contextualized CHAMPION2 trial package of maternal and child health (being implemented in rural villages of Satna district, India) by assessing the quality of, and the factors associated with antenatal care (ANC) provision across four aspects of care and exploring reasons if uptake of care was inadequate.

**Methods:**

We conducted a cross-sectional study in 50 of 196 villages in the CHAMPION2 cluster randomized trial in Satna district, Madhya Pradesh, India before randomization. We interviewed 792 women, who were eligible for the trial and had given birth in the previous two years from the interview date. We assessed the quality of ANC provision across four aspects of care (i.e., skilled care, timeliness (ANC in first trimester), number of ANC visits (at least four), and content of care) and explored women’s reasons if the uptake of care was inadequate. The quality of ANC provision was considered ‘adequate’ if all the four aspects of care were judged sufficient. We conducted logistic regression analyses to determine the socio-demographic factors associated with the adequate quality of ANC provision.

**Results:**

Only 21.2% of women received ANC provision of ‘adequate’ quality (skilled care: 98.9%, timeliness: 75.3%, minimum four ANCs: 73.5%, and appropriate content of care: 28.3%). The inadequate quality was primarily due to inappropriate content of care, particularly poor compliance with iron-folate intake for at least 100 days and no counseling by healthcare providers on key ANC issues. The odds of receiving adequate quality ANC were increased when either the woman or husband was educated to at least high school level.

**Conclusions:**

The quality of ANC provision in the study setting was inadequate. The quality of care was emphasized in refresher training for nurses in the CHAMPION2 trial and health promotion, demand generation, and community mobilization activities were locally contextualized.

**Supplementary Information:**

The online version contains supplementary material available at 10.1186/s12884-026-08925-5.

## Background

In the last two decades, India witnessed a significant drop in maternal (384 to 97 per 100,000 livebirths from 2000 to 2020) and neonatal (38 to 23·5 per 1000 livebirths from 2000 to 2017) mortality rates [1–4]. This is mainly attributed to substantial improvement in the coverage of essential maternal and child health services [5, 6] and various incentive-driven strategic initiatives under the National Health Mission [1, 2]. However, the magnitude of the reductions has been below that predicted [3, 7]. Additionally, there have been growing inequalities in these mortality rates within and across the Indian states [3, 7]. If current mortality trends continue, India may not achieve the Sustainable Development Goal (SDG) targets for maternal (<70/100,000 livebirths) and neonatal (<12/1000 livebirths) mortality [8] by 2030 without further interventions particularly in poorly performing states [3, 7].

According to a pooled analysis of nationally representative surveys, 78.1% of maternal deaths in India in women who planned to deliver at a health facility occurred in a facility and 40.6% of maternal deaths had complications before the onset of labor [7]. Obstetric hemorrhage (47.2%), pregnancy-related infections (12%), and hypertensive disorders of pregnancy (6.7%) were the leading causes of maternal deaths, indicating delayed or missed diagnosis and/or poor management of medical conditions during antenatal care (ANC) [7].

According to a comprehensive district-level analysis of neonatal mortality trends in India, 80% of neonatal deaths occurred within 7 days of birth [3]. Low birth weight and short gestation (child and maternal malnutrition) were the predominant risk factors to which 82.8% (95% CI: 77.6–88.4) of the neonatal deaths could be attributed [3]. In India (and low- and middle-income countries, LMICs), poor quality of care was an important driver of excess mortality (i.e., mortality more than predicted from the reference case fatality level) in 61% of neonatal conditions [9]. This excess mortality may be reduced by improving the quality of healthcare across the continuum of care from pregnancy to delivery [9, 10].

Timely and adequate ANC is a key element of continuum of care, supporting early complication management and sensitizing women about danger signs, breastfeeding, and institutional delivery.

Evidence shows that with adequate coverage and quality of maternal–newborn care, resource-limited LMICs like India could avert 71% of neonatal and 54% of maternal deaths [11]. Previous Indian studies have highlighted the poor quality of maternal and child healthcare [12–21]. Additionally, the quality of care received is associated with women’s socio-demographic factors like age, caste category, literacy, and socio-economic status [12–21]. To further reduce maternal and newborn mortality in India, improving quality of care while considering socio-demographic factors is critical [16, 22].

Given the wide interstate variations in ANC quality [17] and maternal deaths [7], state-specific and socio-culturally contextualized strategies are needed [23, 24]. To inform such strategies in Madhya Pradesh, the CHAMPION2 trial was carried out in Satna district, testing a maternal–child health intervention package (community health promotion and medical provision) [25]. This cluster-randomized trial assigned 196 villages (clusters) to either the health (CHAMPION2 for pregnant women) or education (STRIPES2 (Support To Rural India’s Public Education) for early primary school children) intervention. Villages receiving the health intervention were controls for the education intervention, and vice versa. Further trial details are published elsewhere [25].

Madhya Pradesh has high maternal (173/100,000 live births) and neonatal (29/1000 live births) mortality [4, 26]. At the trial’s planning stage, no studies beyond a secondary analysis of NFHS-4 (National Family Health Survey-4 2015–16) [17] comprehensively assessed ANC quality to guide alignment of the RMNCH+A (Reproductive, Maternal, Newborn, Child, and Adolescent Health) strategy [27] within the local context. Since ANC quality is critical for intervention success and for contextualizing (i.e., adapting the intervention to fit the specific social, cultural, and economic conditions of the target population) the CHAMPION2 interventions package [25], we conducted a cross-sectional study with the following objectives:

1. To assess the quality of provision of ANC in rural villages of Satna district using four aspects of care (i.e., skilled care, timeliness, number and content of ANCs) [28]
2. To explore reasons given by women where uptake of care was inadequate.
3. To investigate the factors associated with the quality of provision of ANC.
4. To help inform the development of an evidence-based contextualized package of maternal and child health interventions for the CHAMPION2 trial [25].

## Methods

### Study setting

Satna is one of the 55 districts in Madhya Pradesh state with an estimated population of about 2.62 million. Rural villages constitute 79% of the district’s population with a predominantly agrarian economy. There are 10 *Tehsils* (administrative subdivision in a district) and 2125 villages in Satna district. The public healthcare system in Satna consisted of 309 sub-centres, 47 primary health centres (PHCs), seven community health centres (CHCs), two civil hospitals (CH), and one district hospital (DH) [29]. At village level, Auxiliary Nurse Midwives (ANM) and Accredited Social Health Activists (ASHA) render maternal and child health services under the RMNCH+A strategy, which is a key component of the National Health Mission [27].

### Design and participants

This cross-sectional study was conducted in 50 of the 196 villages that were subsequently randomized to receive either the CHAMPION2 (health) or the STRIPES2 (education) intervention in the trial [25]. The 50 villages were selected using stratified random sampling with stratification according to the nearest CHC to the village. In the 196 trial villages, a woman was considered eligible for the trial if she was a resident of the village, married, aged less than 50 years, neither she nor her husband had a family planning operation (i.e. tubectomy or vasectomy) and consented to participate. As part of the trial baseline enumeration in 2017 (prior to selection of villages for this study) potentially eligible women had been asked whether they had given birth in the previous year (Supplementary file 1). Approximately one year later we returned to all women in the 50 selected villages, who were both eligible for the trial and had earlier reported a birth in the year prior to enumeration. These women were asked to participate in the present study and then asked questions relating to their most recent birth. This most recent birth must have been in the two years preceding the date of interview for this study: but could have occurred after the one reported at enumeration. 

### Sample size and sampling framework

Pragmatically we aimed to include all the women eligible for this study in 50 randomly selected villages. 

### Assessment of quality of provision of ANC 

Assessment of the quality of care is complex and requires a multidimensional approach. Using a system-based model, the quality of care is assessed under three domains i.e., structure, process, and outcome [30]. The World Health Organization (WHO) further expanded the ‘process’ domain into two linked dimensions of ‘provision of care’ and ‘experience of care’ [31]. Heredia-Pi et al. [28] used four aspects of ‘provision of care’ to assess the adequacy of ANC in Mexico i.e., skilled care, timeliness, sufficiency, and appropriate content of care.

In this study, to inform the development of a contextualized evidence-based package of maternal and child health interventions in the CHAMPION2 trial [25], we focused on ‘provision of care’ i.e., skilled care, timeliness, number of ANCs, and appropriate content of care to assess the quality of ANC [28]. These four aspects were defined as:

1. Skilled care: received ANC by a doctor, ANM, nurse or midwife.
2. Timeliness: received first ANC visit in the first trimester of pregnancy.
3. Number of ANCs: at least four ANCs completed during pregnancy.
4. Content of ANC: Based on the criteria used by previous studies [17, 28], we considered ANC content as “appropriate” if at least 7 out of 8 of the following procedures or processes of care were given to a woman: (1) measurement of weight; (2) blood pressure; (3) urine test; (4) abdominal examination (with or without a machine); (5) tetanus vaccination; (6) blood test (to assess anemia and possibly parasite and other infections); (7) consumed iron-folic acid (IFA) for at least 100 days; (8) counseling on healthy eating, personal hygiene, adequate sleep and rest during the day, and not smoking/drinking alcohol.

### Outcome measure

The quality of provision of ANC was considered as ‘adequate’ if a woman satisfied all the above-mentioned four aspects of care.

### Data collection

We developed a structured questionnaire based on the latest WHO guidelines and manuals by the National Health Mission (NHM), India to assess the ANC details and to explore the factors associated with access to ANC (Supplementary file 2).

Two language experts translated the English questionnaire to Hindi and SS back translated to English. SK, HR, and SS trained 12 enumerators over a period of 5 days for data collection using the Hindi version of the questionnaire. All the enumerators pilot tested the questionnaire in two non-study villages. Each interview lasted for 30-40 minutes. The trained enumerators conducted face-to-face interviews of eligible women using the structured questionnaire from November to December 2018. 

Two data entry operators independently entered the data from the completed questionnaires into a data management system set-up by Sealed Envelope Ltd, London (www.sealedenvelope.com). A data entry supervisor reviewed and resolved any discrepancies in the entered data. 

### Statistical analysis

We used descriptive statistics to summarize the sample characteristics. 95% confidence intervals (CI) for proportions were constructed using robust (Huber-White) standard errors that allowed for clustering.

Logistic regression models, with robust (Huber-White) standard errors that allowed for clustering, were used to separately relate woman’s age at interview (continuous), caste (4-level categorical: scheduled caste, scheduled tribe, other backward class, general category) and education status (5-level categorical: No schooling, primary, middle, high school/higher secondary, graduate and above) to a binary variable indicating adequate quality of ANC provision.

In India, both women’s and their husbands’ education levels are strongly associated with the quality of ANC provision [17–20]. Women and/or husband with better education level are more likely receive adequate quality of ANC provision [17–20]. Three measures of education status were considered (i) woman’s education, (ii) husband’s education and (iii) maximal education (highest of i) and ii)). Participants with missing education status were omitted from models. *P*-values from Wald tests (with joint tests for categorical variables) and from trend tests (education level variables only) were computed.

Simultaneous inclusion of woman’s and husband’s education in multivariable models induces collinearity because they are highly associated with one another. Accordingly, we first fitted three multivariable logistic regression models, each including age, caste, and one of the three education variables. Each of these was then extended by including a three-level categorical variable indicating the relative education of the couple (husband more educated, same level of education, wife more educated). Participants missing either maternal or husband’s education status were omitted from models. Robust standard errors that allowed for clustering were used in all models. *P*-values from Wald tests (with joint tests for categorical variables) and from trend tests (education level variables only) were computed. The fits of nested models (i.e., pairs of models where one model contains a subset of the predictor variables in the other model but no additional variables) were compared using Wald tests. The fits of non-nested models (i.e., pairs of models where each model contains at least one predictor variable not included in the other model) were compared using the Quasi-Likelihood information criteria (a variant of Akaike’s information criterion suitable for use with robust standard errors) [32, 33].

Stata version 16 (StataCorp. 2019. Stata Statistical Software: Release 16. College Station, TX: StataCorp LLC) was used to perform the analyses.

## Results

Of the 1054 eligible women identified in the 50 villages, 797 completed the interview, 128 were not in the village, 115 gave birth more than 2 years before the interview date, 13 did not give consent, and one was a duplicate. Of these 797 women, four did not deliver a live-birth and one reported data on a birth born more than two years before the survey, so the analysis is restricted to 792 women. The mean age of these women was 24.3 ( SD 3.5) years and almost all of them were Hindus by religion (99.6%). Only 14.8% women and 9.2% of husbands had no schooling. Nearly half of them belonged to either Scheduled Caste or Scheduled Tribe (Table 1).

**Table 1:** Socio-demographic profile of women in rural villages of Satna district, Madhya Pradesh, India (*N* = 792)

Characteristic		Mean (SD)	Min, Max
Woman’s age		24.3 (3.5)	18, 42
		***N***	**%**
Education status	No schooling	117	14.8
	Primary	114	14.4
	Middle School	301	38.0
	High School	179	22.6
	Higher-Secondary School	53	6.7
	Graduate	18	2.3
	Postgraduate	9	1.1
	Not Known	1	0.1
Religion	Hindu	789	99.6
	Muslim	3	0.4
Caste-category	Scheduled Caste	164	20.7
	Scheduled Tribe	222	28.0
	Other Backward Class	315	39.8
	General category	91	11.5
Husband’s education	No schooling	73	9.2
	Primary	138	17.4
	Middle School	263	33.2
	High School	193	24.4
	Higher Secondary School	75	9.5
	Graduate	33	4.2
	Postgraduate	12	1.5
	Not Known	5	0.6

Most of the women (770, 97.2%) sought ANC from the public (government) healthcare facilities. Table 2 shows the various aspects of quality of ANC provision. Almost all women received skilled health care with ANC starting in the first trimester in around three-quarters of cases. 73.5% of women had at least four ANC visits (but only 98 (12.4%) had at least eight visits). However, only 28.3% received ANC with sufficient procedures and processes of care. This low proportion was the primary reason why only 21.2% (95% CI 16.8, 25.6) of the women had ‘adequate’ quality of ANC provision (i.e., received ANC from a skilled health personnel, first ANC visit within the first trimester, completed at least 4 ANC visits and with appropriate ANC content). The following proportions of women received the various procedures or processes of care received during ANC: weight measured (95.2%), blood pressure recorded (92.3%), abdomen examined (with or without a machine) (72.9%), received tetanus vaccination (98.6%), urine tested (68.3%), blood tested (89.3%), consumed IFA for at least 100 days (17.0%) and counseled on key ANC issues (30.4%).

**Table 2:** Quality of Antenatal Care (ANC) provision to women in rural villages of Satna district, Madhya Pradesh, India (*N* = 792)

Aspect of quality of ANC provision	Number	% (95% CI)
i. Skilled health care	783	98.9 (97.9, 99.8)
ii. Timeliness (ANC in first trimester)	596	75.3 (71.1, 79.4)
iii. Number of ANCs (at least four visits)	582	73.5 (69.1, 77.8)
iv. Appropriateness in ANC content	224	28.3 (23.3, 33.2)
**Overall adequacy of quality of ANC provision** **(all of i. to iv. above)**	**168**	**21.2 (16.8, 25.6)**

Only two women didn’t seek any ANC from a skilled health person. Of these, one woman stated that the nearest health facility was too far away, and a vehicle was not available. The other woman stated that she was in a different town for work. The following reasons were given by the women who did not complete their first ANC within the first trimester and/or attended less than four ANC visits: they didn’t think they needed early or more ANC (51.0% and 63.8%, respectively), they didn’t know they could go (37.6% and 18.1%), they didn’t have time (11.3% and 17.6%), the health facility was too far away (9.8% and 15.7%) and they were out of the village (13.9% and 13.3%). The reasons were not mutually exclusive.

Poor compliance with 100 days of IFA tablet intake was mainly attributed to the fact that women were not given the IFA supplements (34.0%) and did not feel well when they started taking them (42.0%). Other reasons for poor compliance with IFA intake were as follows (not mutually exclusive): the woman was not told to take IFA for at least 100 days (14.7%), she received the tablets but forgot to take them (14.0%), she did not feel IFA intake was necessary (8.1%), and she reported that she had a previous bad experience with IFA intake (5.0%).

Table 3 shows the unadjusted associations between socio-demographic characteristics and the binary ‘quality of ANC provision’ indicator. There was no statistically significant evidence of an association with age but there was borderline statistically significant evidence of an association with caste, and strong evidence of an association with education, whether that of the woman or husband. In the analysis of the maximal (amongst woman and husband) education level, the odds of adequate quality of ANC provision were similar in the three lowest education categories and markedly increased in those receiving at least high school education.

**Table 3:** Unadjusted associations between socio-demographic characteristics of women and adequate quality of ANC provision in rural villages of Satna district, Madhya Pradesh, India (*N* = 792)

Socio-demographic characteristic	Category	Adequate quality of provision of ANC				Odds ratio(95% CI)	*p*-value
		Yes (*N*=168)		No (*N*=624)			
		Mean	SD	Mean	SD		
Woman’s age (years)		24.4	3.3	24.3	3.5	1.01^1^ (0.97, 1.05)	0.72
		***N***	**%**	***N***	**%**		
Caste-category	Scheduled Caste	40	23.8	124	19.9	1	0.0591 (joint test)
	Scheduled Tribe	31	18.5	191	30.6	0.50 (0.29, 0.88)	
	Other Backward Class	71	42.3	244	39.1	0.90 (0.60, 1.35)	
	General category	26	15.5	65	10.4	1.24 (0.65, 2.35)	
Woman’s education status	No schooling	17	10.1	100	16.0	0.61 (0.35, 1.04)	0.0084 (joint test) 0.0007 (trend test)
	Primary	13	7.7	101	16.2	0.46 (0.25, 0.85)	
	Middle	66	39.3	235	37.7	1	
	High/higher secondary	60	35.7	172	27.6	1.24 (0.88, 1.75)	
	Graduate and above	12	7.1	15	2.4	2.85 (1.09, 7.44)	
	Missing^2^	0	0.0	1	0.16		
Husband’s education status	No schooling	10	6.0	63	10.1	0.75 (0.36, 1.57)	0.0001 (joint test) 0.0029 (trend test)
	Primary	24	14.3	114	18.3	0.99 (0.59, 1.66)	
	Middle	46	27.4	217	34.8	1	
	High/higher secondary	72	42.9	196	31.4	1.73 (1.24, 2.42)	
	Graduate and above	16	9.5	29	4.6	2.60 (1.46, 4.64)	
	Missing^2^	0	0.0	5	0.8		
Maximal education status	No schooling	6	3.6	32	5.1	1.11 (0.45, 2.75)	< 0.0001 (joint test) 0.0138 (trend test)
	Primary	11	6.5	66	10.6	0.99 (0.50, 1.95)	
	Middle	40	23.8	237	38.0	1	
	High/higher secondary	91	54.2	246	39.4	2.19 (1.51, 3.18)	
	Graduate and above	20	11.9	37	5.9	3.20 (1.69, 6.08)	
	Missing^2^	0	0.0	6	1.0		

^1^ The odds ratio for age relates to a one-year increase in age

^2^ Omitted from analyses

In the education adjusted analyses relating socio-demographic characteristics to the binary ‘quality of ANC provision’ indicator there was no statistically significant evidence of an association with age or with caste after adjustment for education, whether that of the woman, husband, or the maximal level. As measured using the quasi-likelihood information criteria (QIC), the best fitting of the adjusted models included the maximal level of education along with age and caste (Table 4). As in the unadjusted analysis, the odds of the adequate quality of ANC provision were similar in the three lowest education categories and markedly increased in those receiving at least high school education. In an extended model there was no evidence that odds of adequate quality of ANC provision differed if just the woman or husband had received the maximal education level (odds ratio = 1.02 (95% CI 0.71, 1.49) for the comparison between families where just the woman had reached this level and families where both had; odds ratio = 0.91 (95% CI 0.60, 1.40) for the comparison between families where just the husband had reached this level and families where both had). Models where just the woman’s education (QIC = 812.375) and just the husband’s education (QIC = 814.531) was included fitted less well than the model with their maximal level of education (QIC = 808.215). For both such models, extensions provided some evidence that where the other partner had a higher level of education the odds of adequate quality of ANC provision were increased, providing additional support for the hypothesis that is the maximal level of education among the couple that is key.

**Table 4:** Mutually adjusted associations between socio-demographic characteristics of women and adequate quality of ANC provision in rural villages of Satna district, Madhya Pradesh, India (*N* = 786)

Socio-demographic characteristic	Category	Odds ratio (95% CI)	*p*-value
Woman’s age (years)		1.02 (0.98, 1.07)	0.28
Caste-category	Scheduled Caste	1	0.63 (joint test)
	Scheduled Tribe	0.67 (0.36, 1.25)	
	Other Backward Class	0.92 (0.60, 1.40)	
	General category	0.98 (0.53, 1.81)	
Maximal education status	No schooling	1.11 (0.40, 3.04)	0.0041 (joint test) 0.0537 (trend test)
	Primary	1.05 (0.53, 2.08)	
	Middle	1	
	High/higher secondary	2.06 (1.35, 3.13)	
	Graduate and above	2.84 (1.47, 5.48)	

## Discussion

This study provides insights concerning the quality of ANC provision across four aspects of process of care in a high priority district of Madhya Pradesh, India. The following key findings emerged from our analysis. First, only 21.2% of women in rural villages of Satna district received ANC that was of ‘adequate’ quality. Second, inadequate quality of ANC provision was primarily due to inappropriate ANC content (i.e., various procedures and processes) delivered by care providers. Notably, women’s poor compliance with IFA intake for at least 100 days and lack of counseling by care providers on the key aspects of ANC emerged as the main issues. Women with no early and/or fewer than four ANCs thought they didn’t need ANC sufficiently early or frequently, or reported not knowing that they could access ANC. Third, a woman educated to at least high school level (or whose husband had been) was more likely to receive adequate quality of ANC provision than a woman from a family when neither had been educated to this level.

In some previous work [5, 18–20], ‘full ANC’ was declared when a woman had four or more ANC visits, received at least one tetanus injection, and took IFA for 100 or more days. However, unlike the present study, such ‘full ANC’ criteria do not consider ‘skilled care’, ‘timeliness’, and ‘appropriate content of care’ while assessing the quality of provision of ANC, which is an important limitation. Similar to the present study, a secondary analysis of NFHS-4 data using all the four aspects of care reported that very low proportion (about 16%) of women in Madhya Pradesh received adequate quality of ANC provision [17]. As in this study, appropriate ANC content was the least satisfied aspect of care provision in other studies in India [17, 21]. Although nearly three-quarters of women had at least 4 ANC visits, only 28.2% of women received ANC with appropriate content, suggesting missed opportunities by the healthcare providers or women.

Pregnant women’s poor compliance to IFA intake for at least 100 days is a pan-India issue (30.3% in NFHS-4 (2015-16) and 54% in NFHS-5 (2019-21)) [6], with compliance worse in rural areas (40.2% in NFHS-5 (2019-21)) [6]. In the latest nationwide survey (NFHS-5), compliance to IFA intake during pregnancy in Satna district was 41.3% (urban and rural combined) [34], which is much higher than in our study (where all villages are rural) (17%). Our study findings reveal that poor IFA compliance in rural Satna district (17%) stems from both supply-side and demand-side barriers. Supply-side issues were predominant: 34% of women reported not receiving IFA supplements, reflecting stock-outs and distribution lapses that align with nationwide evidence [35] showing only 213 of 704 Indian districts maintain adequate year-round IFA supplies due to poor forecasting, inventory management, and distribution systems. In our study demand-side factors independently contributed to non-compliance: 42% of women discontinued due to side effects, while others cited inadequate counseling on the 100-day requirement, forgetfulness, or perceptions that IFA was unnecessary. These findings underscore that low IFA adherence results from systemic supply chain failures compounded by inadequate counselling and support, rather than individual behavioral choices alone. Addressing both strengthened district-level logistics and comprehensive counseling is essential to improve IFA availability and consumption in rural settings [35, 36]. Gestational anemia is a major public health issue in Madhya Pradesh (prevalence of 52.8%) [6] and postpartum hemorrhage is the most common cause of maternal mortality in India [7], co-existing anemia (particularly ‘severe’ anemia) increases the risk of death or maternal near miss [37–39]. Therefore, addressing these dual barriers of IFA compliance in pregnancy is necessary in order to improve maternal and child health outcomes. In addition, lack of counseling on key ANC related issues was another major issue in this and one other study [21] during ANC, this being vital in empowering women to take necessary actions for a positive pregnancy experience and to prevent adverse birth outcomes [40].

In this and other studies [16–19], at least four ANC visits during pregnancy was considered as adequate according to the national guidelines. The new WHO ANC model recommends at least eight ANC visits during pregnancy [40]. However, based on the inadequacy in quality of ANC provision in this and other studies in India [17, 21], increasing the minimum number of ANC visits may not translate into the desired health outcomes unless the quality of ANC provision is addressed [41].

In our study, woman’s age (as a continuous variable) showed a small and non-statistically significant association with receiving ANC of adequate quality. Older women may have had more babies (the fact that we don’t consider parity in our analysis is a limitation) and could impact on our result in several ways. Previous studies in India reported that women of adolescent age were less likely to receive ANC of adequate quality [17, 18]. Similarly, when compared to primigravidae, multiparous women were less likely to receive ANC of adequate quality [17, 18]. Miteniece et al. [42], reported increased confidence from previous pregnancy and childbirth experience, constraints of time and resources, poor prior experience with the health system and financial barriers as potential reasons for inadequate ANC quality provision in multiparous women.

Regarding woman’s caste, we did see some evidence (albeit not formally statistically significant) of an association, but adjustment for education renders the effects smaller and not statistically significant, suggesting that education plays a mediating role here. In this study, woman/husband with higher education were more likely to receive ANC of adequate quality. Previous Indian studies have highlighted those women with higher education status (or whose husband has higher education status) are more likely to receive ANC of adequate quality [17, 18, 43]. According to Das et al., women and/or their husband who had a higher level of education were more likely to seek ANC from competent health professionals when compared to those with lower educational levels [44]. Higher education levels may empower women and/or their husbands to demand better care as well as improving their comprehension of health information and their ability to communicate with providers and navigate the healthcare system. A secondary analysis of NFHS-4 data [43] from five Indian states including Madhya Pradesh found evidence of a statistical interaction between a women’s primary education and their partners’ higher education indicative of a ‘substitution effect’ such that a husbands’ higher education compensated for women’s lower education in influencing ANC service use. Another secondary analysis of NFHS-5 data reported that quality of maternal and newborn care in India displayed a greater variation between smaller areas within districts [45]. One of the mechanisms could be clustering of these key socio-demographic characteristics (education, wealth, caste, etc.), which tend to cluster more densely at the household or small area level [45]. Despite the statistical significance of the association between higher education and receipt of adequate ANC, only around a third of women (111/394) who had (or whose husband had) been educated to at least High/Higher secondary level received ANC provision of adequate quality, suggesting that any intervention aimed at improving ANC should not be restricted to those with lower levels of education.

Contextualizing the CHAMPION2 intervention: The maternal and child health intervention package in the CHAMPION2 trial [25] consisted of community health promotion, community mobilization with women’s groups in the form of participatory learning and action (PLA), provision of nurse-led fixed-day services and participatory discussion groups (PDGs) with pregnant women, and facilitation of referrals of mothers and neonates to CHCs or CHs. After randomization of villages in the CHAMPION2 trial, the intervention team conducted quantitative and qualitative appraisals in the intervention villages to help identify key areas in need of improvement through interviews and focus groups with local women, healthcare providers and village elders. These appraisals by the intervention team and the findings of this study substantiated our approach in contextualizing key components of the CHAMPION2 intervention. For health promotion and demand generation, a health awareness campaign was launched in the villages, targeting all the community members to promote maternal and neonatal health knowledge. Focus groups and *Nukkad Nataks* (traditional village-level street plays conveying health messages) were conducted for the campaign, which were adapted based on local customs to convey important maternal and child health messages to communities. Further, we planned to allow all the village women to participate in the PLA sessions focusing on improving women’s health knowledge, encouraging greater use of available services, other key aspects of maternal and child health, and providing women with a forum to discuss solutions to important maternal and neonatal health issues.

As a part of the CHAMPION2 intervention, refresher training of nurses was conducted to improve the implementation of various components of ANC content. Nurses were also trained to conduct participatory discussion groups (PDGs) with pregnant women during ANC sessions. In refresher training for nurses, we placed emphasis on all the processes/procedures of ANC, ensuring an adequate IFA supply and counseling on various key ANC issues including IFA compliance (i.e., explaining and reassuring regarding initial temporary side effects and emphasizing the benefits of daily intake). PDGs included various key topics including the importance of ANC, diet in pregnancy and after delivery, importance of IFA intake, etc. Nurses were advised to share the relevant information with PLA teams based on their experiences or events during ANC and PDG sessions so that these could be discussed at the next PLA session with village women.

### Strength and limitations

The study’s main strength was the comprehensive assessment of the quality of ANC provision across all four aspects of care in the eligible population. However, the assessment relied on women’s recall, and we could not verify all responses. Also, data on women’s parity were not collected, and it is possible that parity may influence ANC quality provision. This study also excluded women who had abortion/s or stillbirth/s without any live birth/s in the two years preceding the survey in order to avoid distress potentially caused by discussing such sensitive issues in rural village settings. Consequently, the findings may not be generalizable to these women. Future research should strategically engage women with a history of abortion or stillbirth and collect parity data to provide a more comprehensive understanding of ANC quality. Lastly, this study focused only on the quality of ANC provision as insufficient information was available for postnatal and neonatal care provision across all the four aspects of care.

## Conclusions

Overall, the quality of ANC provision in rural villages of Satna district was inadequate, which was primarily due to inappropriate content of ANC. Women or their husbands having education to at least high school level increased the odds of receiving ANC of adequate quality. The findings of this study substantiated our approach in contextualizing key components of the maternal and child health intervention package in the CHAMPION2 cluster randomized trial. The quality of ANC has many components, the content of ANC being one. It is likely that inadequate quality of ANC provision in similar settings will also be related to the content of ANC, and that contextualized health promotion, demand generation, and community mobilization activities as well as refresher training will be helpful in improving the quality of ANC provision in similar rural settings. Based on our study findings, there is an urgent need to improve the overall quality of ANC, particularly the content of ANC, in rural villages of Satna district. The broader body of evidence [9–11] indicates that strengthening ANC quality has the potential to reduce maternal and neonatal morbidity and mortality in this setting, but further evidence from large randomized controlled trials is required to confirm this.

## Supplementary Information

Supplementary Material 1.

Supplementary Material 2.

## Data Availability

The datasets generated during this study are not publicly available but are available from Ila Fazzio (if@effint.org) on reasonable request.

## Abbreviations

ANC: Antenatal care
ANM: Auxiliary Nurse Midwife
ASHA: Accredited Social Health Activist
CH: Civil hospital
CHAMPION2: Community health promotion and medical provision and impact on neonates
CHC: Community health center
CI: Confidence interval
DH: District hospital
EAG: Empowered Action Group
LMIC: Low and middle-income country
NHM: National Health Mission
NFHS: National Family Health Survey
PDG: Participatory discussion group
PHC: Primary health centre
PLA: Participatory Learning and Action
QIC: Quasi-likelihood information criteria
RMNCH+A: Reproductive, Maternal, Newborn, Child, and Adolescent Health
SDG: Sustainable Development Goals
STRIPES2: Support to rural India’s public education system and impact on numeracy and literacy scores
WHO: World Health Organization
