# Supplementary material for: Quality of antenatal care provision in rural villages of Satna district, Madhya Pradesh, India: a quantitative formative study to help the development of an evidence-based contextualized complex health intervention of the CHAMPION2 cluster randomized trial
Source: BMC Pregnancy Childbirth. 2026 Mar 23;26:593. doi: 10.1186/s12884-026-08925-5 (PMC13224558; doi:10.1186/s12884-026-08925-5)
Supplement: Supplementary file 1 — Supplementary Material 1. [file 12884_2026_8925_MOESM1_ESM.pdf]

Effective Intervention | GH Training and Consulting

MP trial CHAMPION 2

Woman Enumeration WE

VILLAGE ID ☐☐☐

HOUSE ID ☐☐ - ☐☐

WOMAN ID ☐☐

You have completed the HOUSE form with the HEAD of the house. Now you should ask to talk with those women who are RESIDENT, MARRIED, and UNDER 50 years old.

	QUESTIONS	ANSWERS	SKIP
1	Read the CONSENT REQUEST FOR THE WOMAN	IF SHE CONSENTS, KINDLY ASK HER TO PLEASE SIGN/THUMBPRINT BELOW:<br>____________________<br>☐<br>SIGNATURE OR THUMBPRINT .... 1<br>SHE DOES NOT CONSENT 0	→ NEXT LINE<br>→ THANKS/FINISH
2	Are you resident in this village?	NO .... 0<br>YES.. 1	→ THANKS/FINISH<br>→ NEXT LINE
3	What is your age?<br><br>Try your best to obtain her age.<br>If she does not know enter 88	YEARS OLD ☐☐	IF WOMAN IS 50 OR MORE THANKS/FINISH
4	Have you or your husband had a family planning operation?	NO .... 0<br>YES.. 1	→ NEXT LINE<br>→ THANKS/FINISH
5	What is your name?		
6	What is your surname?		
7	What is your husband's name?		
8	What is your husband's surname?		
9	Have you ever attended school?	NO .... 0<br>YES.. 1	→ 11<br>→ NEXT LINE
10	What is the highest grade you completed?<br><br>If she has not completed grade 1 enter 00<br>If she does not know enter 88	GRADE ☐☐	
11	Ask her to PLEASE read the sentence in the reading card.	CANNOT READ AT ALL .... 1<br>READ ONLY PART OF IT .... 2<br>READ WHOLE SENTENCE 3	
12	What is your family's caste?	SCHEDULE CASTE .... 1<br>SCHEDULE TRIBE .... 2<br>OTHER BACKWARD CASTE .... 3<br>FORWARD CASTE 4	

MP trial CHAMPION 2 - Woman Enumeration Form

**13**	What is your religion?	HINDU .... 1<br>MUSLIM .... 2<br>CHRISTIAN .... 3<br>BUDDHIST .... 4<br>SIKH .... 5<br>OTHER .... 6<br><br>specify ______________________________	
**14**	How old is your husband?<br><br>If she does not know enter 88	YEARS OLD ☐☐	
**15**	Would he please read this sentence to me?<br>SHOW CARD TO THE HUSBAND OF RESPONDENT.	HE IS NOT PRESENT.................................... 0<br>CANNOT READ AT ALL .... 1<br>READ ONLY PART OF THE SENTENCE ..... 2<br>READ WHOLE SENTENCE 3	
**16**	Has your husband ever attended school?	NO .... 0<br>YES... 1<br>DON'T KNOW 8	→ **18**<br>→ **NEXT LINE**<br>→ **18**
**17**	What is the highest grade he completed?<br><br>If he has not completed grade 1 record 00<br>If they do not know record 88	GRADE ☐☐	
**18**	Now I would like to talk about pregnancy and babies<br>Have you ever been pregnant in the past (even if lost baby)?	NO .... 0<br>YES... 1	→ **24**<br>→ **NEXT LINE**
**19**	Have you ever given birth (even if baby died)?<br>If so, how many times?	NUMBER OF TIMES GAVE BIRTH ☐☐	→ **IF 00 GO TO 24**
**20**	Have you ever given birth to a baby who was born alive but later died? If so, how many times?	BABIES BORN ALIVE WHO DIED ☐☐	
**21**	In the last 12 months, have you given birth to a baby?	NO .... 0<br>YES... 1	→ **24**
**22**	Was s/he born alive but later died?	NO .... 0<br>YES... 1	→ **24**
**23**	Did s/he die in the first month?	NO .... 0<br>YES .... 1<br>DON'T KNOW 8	
**24**	Are you pregnant now?	NO .... 0<br>YES .... 1<br>DON'T KNOW 8	

Thanks and finish the interview

Interviewer signature .... ID ☐☐

Supervisor signature .... ID ☐☐
