# Supplementary material for: Quality of antenatal care provision in rural villages of Satna district, Madhya Pradesh, India: a quantitative formative study to help the development of an evidence-based contextualized complex health intervention of the CHAMPION2 cluster randomized trial
Source: BMC Pregnancy Childbirth. 2026 Mar 23;26:593. doi: 10.1186/s12884-026-08925-5 (PMC13224558; doi:10.1186/s12884-026-08925-5)
Supplement: Supplementary file 2 — Supplementary Material 2. [file 12884_2026_8925_MOESM2_ESM.pdf]

MP trial CHAMPION 2

KAP survey

Tehsil:
Village ID:
Village:
Cluster ID:
Household ID:
Woman ID:
Woman´s name:
Husband's name:
Head of the household:

INTERVIEW DATE

DAY	MONTH	YEAR	HOURS	MINUTES
☐☐	☐☐	☐☐☐☐	: ☐☐	☐☐

**INTRODUCTION AND CONSENT CONFIRMATION FOR KAP**
READ THE EXPLANATION ABOUT THE SURVEY:<br><br>*My name is ___________________________from GH Training and Consulting based in Satna. You previously agreed to be part of a four-year study in rural villages in Satna district in which your village will receive one of two possible interventions: either an after-school programme aiming to increase the levels of education in primary school children or a health promotion and support programme, aiming to improve the health of mothers and new born babies. This study is organised and financed by Effective Intervention.*<br><br>*As part of this work we are conducting a survey to understand the knowledge, attitude and practices related to pregnancy and child birth. You have been selected for the interview as you gave birth recently. The information you give will help us design the programme to improve the health of pregnant women and newborns. Your participation is voluntary and the data provided by you will be kept confidential and will not be discussed with any other individuals*<br><br>*Do you have any questions? (Interviewer: Provide responses to the queries raised by the woman)*<br>*May I begin the interview now?*<br><br>IF SHE DOES NOT CONSENT CIRCLE OPTION 3IN QUESTION 0.1, THANKS AND FINISH INTERVIEW

INTERVIEW STATUS

To be completed before the start of the interview

No	Question	Codes	Skip
0.1	Interview status	Woman was travelling or moved.........0<br>Woman passed away...............................1<br>No one knows that woman ....2<br>Woman did not give consent.................3 ➡<br><br>Woman agreed to be interviewed 4	**Thanks and finish**
0.2	When was the last time you gave birth?	DAY MONTH YEAR<br>[ ][ ] [ ][ ] [ ][ ][ ][ ]<br><br>**IF SHE HAS NOT DELIVERED WITHIN TWO YEARS PRIOR TO THE DATE OF THE KAP INTERVIEW THANKS AND FINISH!** ➡	**Thanks and finish**
0.3	Was any baby from this birth born alive? Did he/she breathe or cry immediately after birth?	No... ....0 ➡<br><br>Yes.. 1	**Thanks and finish**

SECTION 1: ANTENATAL CARE

(Interviewer*: "Now, I would like to ask you some questions about when you were pregnant with the last baby you gave birth to.")*

No	Question	Codes	Skip
1.1	Did you see anyone for antenatal care in your last pregnancy?	No.. 0<br><br>Yes..1 ➡	**1.3**
1.2	Why didn't you receive antenatal care? Please tell me all the reasons…<br><br>Circle all reasons mentioned and **GO TO 1.10**,<br>You should not read the options aloud, but can ask for clarity!	Someone did not allow me to go................ 1<br>I didn't know I could go............................... 2<br>I didn't have money.....................................3<br>I didn't have time...4<br>Health facility was too far .... 5<br>I didn't think ANC was necessary for me…. 6<br>**If others, write:**<br><br>Don't know/ don't remember............... 98	**CIRCLE AND GO TO 1.10**
1.3	Who did you see for antenatal care for in your last pregnancy?<br><br>Circle all persons mentioned,<br>but do NOT prompt with any suggestions!	Doctor .... 1<br>Auxiliary Nurse Midwife......................... 2<br>ASHA.. 3<br>Traditional Birth Attendant ....4<br>Anganwadi worker ....5<br>**If others, write:**<br><br>Don't know/ don't remember................. 98	

**1.4**	Where did you receive antenatal care for your last pregnancy?<br><br>Circle all places mentioned,<br>but do NOT prompt with any suggestions	Government Hospital……………. ……………..1<br>Private Hospital……………………………………. 2<br>Health centre(public)…….…………………….. 3<br>Anganwadi centre ……………………………..… 4<br>Mobile clinic………………………. ……………..…. 5<br>Private clinic in the village …. ………….……. 6<br>At home…………………………..………. ……………. 7<br>Vaccination point in the village ……………. 8<br>**If others, write:**<br><br><br>Don't Know………………………………….………98	
1.5	How many times did you receive antenatal care during your last pregnancy?	Number of times _________<br><br>Don't know……………………98	**IF 4 TIMES OR MORE GO TO 1.7**
1.6	Why didn't you receive more antenatal care?<br>Please tell me all the reasons…<br><br>Circle all reasons mentioned.<br>You should not read the options aloud, but can ask for clarity!	Someone did not allow me to go……………. 1<br>I didn't know I could go…………………………. 2<br>I didn't have money……………………………….3<br>I didn't have time…………………………………..4<br>Health facility was too far …………………….. 5<br>I didn't think ANC was necessary for me.. 6<br>I delivered before my last ANC …………….. 7<br>**If others, write:**	
1.7	What tests, vaccines, exams and counselling were done/given in these ANCs?<br><br>Read the options and circle:<br>0 for No<br>1 for Yes<br>98 for don't know	(see table below)	

**Were you given/tested?**	**NO**	**YES**	**Don't know**
Height	0	1	98
Weight	0	1	98
Blood pressure	0	1	98
Abdominal examination without a machine	0	1	98
Abdominal examination with a machine	0	1	98
Tetanus vaccination (Tdap, Td or TT)	0	1	98
Urine test	0	1	98
Blood test	0	1	98
Counselling on healthy eating	0	1	98
Counselling on personal hygiene	0	1	98
Counselling on adequate sleep and rest during the day	0	1	98
Counselling about not smoking/drinking alcohol	0	1	98

If others, write:

1.8	How many months pregnant were you when you first had an ANC contact?	Months pregnant when 1st had an ANC contact ________<br>Don't know…………………………………. 98	**IF 3 MONTHS OR LESS GO TO 1.10**
1.9	Why didn't you receive antenatal care earlier in your last pregnancy?[mention the number months she said in question 1.8]<br><br>Please tell me all the reasons…<br><br>Circle all reasons mentioned.<br>You should not read the options aloud, but can ask for clarity!	Someone did not allow me to go……………. 1<br>I didn't know I could go…………………………. 2<br>I didn't have money……………………………….3<br>I didn't have time………………………………….4<br>Health facility was too far ……………………... 5<br>I didn't think ANC was necessary for me…. 6<br>**If others, write:**	
1.10	How many check-ups do you think a woman should have with a nurse/doctor while she is pregnant?	Number of check ups _______<br>Don't know ……………………….. ………………. 98	
1.11	In which month of pregnancy do you think a woman should go to a nurse or doctor for her first antenatal check up?	Month pregnant for 1st ANC _______<br>Don't know ……………………….. ………………. 98	
1.12	Did you take IFA (iron) tablets during your pregnancy?<br>Show to the woman a box/image…	Not a single tablet was taken……………. 0<br><br>Yes……………………………………………….1 ➡<br><br>Don't know ………………………………. 98 ➡	**1.14**<br><br>**1.16**
1.13	Why did you not take IFA (iron) tablets at all?<br><br>You should not read the options aloud, but can ask for clarity!<br>Circle all reasons mentioned	I was not told to take any IFA (iron) ……………. 1<br>I was not given any IFA (iron) …………………….. 2<br>I didn't know that I had to take IFA (iron) …… 3<br>**If others, write:**	**CIRCLE AND GO TO 1.16**
1.14	How many IFA (iron) tablets did you take?<br><br>Show the IFA package and ask if it was more or less than one hundred tablets	Less than 100 tablets …………………………. 0<br><br>100 or more tablets ……………………….. 1<br>Don't know …………………………………. 98 ➡	**1.16**
1.15	Why did you not take at least 100 IFA (iron) tablets?<br><br>You should not read the options aloud, but can ask for clarity!<br>Circle all reasons mentioned	I was not told to take at least 100 IFA (iron) …. 1<br>I was not given 100 IFA (iron) ……………………. 2<br>I was not feeling well when started taking the IFA tablets (constipation, vomiting, diarrhoea, black stools) ………………………………………………… 3<br>I received but forgot to take them ……………….. 4<br>I did not feel IF intake was necessary ……………. 5<br>My family member told not to take IFA ……….. 6<br>**If others, write:**	

1.16	When *a woman* is pregnant, what signs or conditions indicate that she needs to seek immediate care?<br><br>Circle all reasons mentioned,<br>but do NOT prompt with any suggestions	When I have…<br>Vaginal bleeding (any amount) .... 1<br>Abdominal pain .... 2<br>Severe headache ....3<br>Convulsion .... 4<br>Blurred vision .... 5<br>Swelling of feet/face/hands .... 6<br>Fever ....7<br>Decreased/no foetal movements .... 8<br>Foul smelling vaginal discharge....................... 9<br>Difficulty seeing at night.................................10<br>Difficulty in emptying the bladder ....11<br>Feeling weak/feeling tired/breathlessness ....12<br>Water leak .... 13<br>**If others, write:**<br><br><br>Don't Know 98	

SECTION 2: DELIVERY

(Interviewer*: "Now, I would like to ask you some questions about your last delivery.")*

No	Question	Codes	Skip
2.1	What type of delivery did you have?	Normal delivery..1<br>C-section (cut in your tummy).................. 2	
2.2	Where was your last delivery?	Hospital(health facility) .... 1<br><br>On the way to the hospital(health facility) 2<br>At home ....3<br>Parent's home .... 4<br>Relative's home ....5<br>Mother in law's home ....6<br>**If other, write:**	(2–6) → **2.5**
2.3	Ask the woman the name and location of the hospital (health facility) where she delivered<br><br>NAME<br><br>LOCATION<br><br>Check what kind of hospital (health facility) it is and circle only one option!	Sub Centre .... 1<br>Primary Health Centre............................... 2<br>Community Health Centre.......................... 3<br>District hospital... 4<br>Civil Hospital.. 5<br>Private hospital/clinic................................. 6<br>NGO facility.. 7<br>**If other, write:**	

2.4	What transport did you use to go to the hospital (health facility) where you delivered?<br><br>Circle only one option	108/Janani express ……………………………………… 1<br>Public transport ………………………………………. 2<br>Private vehicle ………………………………………… 3<br>Walk ……………………………………………………… 4<br>**If other, write:**	**FOR ANY ONE GO TO 2.8**
2.5	Why didn't you deliver in a hospital (health facility)? Please tell me all the reasons…<br><br>Circle all reasons mentioned,<br>but do NOT prompt with any suggestions	Cost too much ……………………………… …………..1<br>Hospital not open ……………………………………… 2<br>Too far / no transportation ………………………… 3<br>Don't trust/poor quality service ……………………4<br>No female provider at facility ………………… …… 5<br>Husband / family did not allow ……………………. 6<br>Not necessary ………………………………………….. 7<br>Not customary …………………………………………..8<br>Not enough time to get to the hospital (Delivered before due date) ……………………………………. 9<br>**If others, write:**	
2.6	Was a safe delivery kit used in your last delivery?<br>Show the safe delivery kit to the woman	No……………………………………………………………0<br>Yes……………………………………………………………1<br>Don't know …………………………………………… 98	
2.7	What was used to cut the cord?	New or sterilised blade.………… ……………….1<br>Knife……………………………………………………….2<br>Scissor……………………………………………………3<br>**If others, write:**<br><br>Don't know ……………………………………………98	
2.8	Who was the principal person who conducted your last delivery?<br><br>Circle only one option	Doctor ……………………………………………………… 1<br>Auxiliary Nurse Midwife……………………… ……. 2<br>ASHA……………………………… …………………… 3<br>Traditional Birth Attendant …………………………4<br>Anganwadi worker …………………………………… 5<br>Relative/Friend (NON TRAINED) ………………… 6<br>Nobody ……………………………………………………. 7<br>**If others, write:**<br><br>Don't know/ don't remember……………………98	

SECTION 3: POSTNATAL CARE

(Interviewer: *"Now I am going to ask you some more detailed questions about the postnatal care that you received.* I will start ask about you and then about your baby*")*

No	Question	Codes	Skip
3.1	Did any nurse/doctor or any other health worker check on your health in the first two days after delivery?	No……………………………………………………0 ➡<br>Yes……………………………………………………1	4.1
3.2	Who checked on your health in the first two days after delivery?<br><br>Circle all persons mentioned,<br>but do NOT prompt with any suggestions	Doctor ……………………………………………… 1<br>Auxiliary Nurse Midwife………. …………………..2<br>ASHA………………………………………………… 3<br>Traditional Birth Attendant ………………………… 4<br>Anganwadi worker ……………………………………5<br>**If others, write:**<br><br>Don't know/ don't remember………………….. 98	
3.3	During these visits were you checked/advised about the following?<br><br>Read the options and circle :<br>0 for No<br>1 for Yes<br>98 for don't know	(see sub-table below)	

	No	Yes	Don't know
Checked for fever after delivery	0	1	98
Examined abdomen	0	1	98
Checked blood pressure	0	1	98
Asked for excessive vaginal bleeding	0	1	98
Asked for fits	0	1	98
Advised about iron tablets (100 IFA)	0	1	98
Advised about nutrition	0	1	98
Advised about exclusive breastfeeding	0	1	98
Advised about family planning	0	1	98

SECTION 4: IMMEDIATE NEWBORN CARE

(Interviewer*: "Now, I would like to ask you some questions about the care of your child after delivery. If you had twins, please refer to the first baby born alive")*

No	Question	Codes	Skip
4.1	Did your baby survive his/her first day of life?	No……………………………………………………… 0 ➡<br>Yes……………………………………………………… 1	**Thanks and finish**
4.2	Was your baby placed on your chest with skin-to-skin contact **immediately** after birth?	No………………………………………………………0<br><br>Yes…………………………………………………… 1<br>Don't know……………………… ….. ….. ……….. 98 ➡	**4.5**

4.3	Why wasn't your baby put on your chest skin-to-skin **immediately** after birth?<br><br>Circle all reasons mentioned	I had a C-section............... .... 1<br>Baby was taken away as s/he was not well .... 2<br>I was not feeling well .... 3<br>Not customary .... 4<br>**If others, write:**<br><br><br>Don't know.. 98	
4.4	Was your baby placed on your chest with skin-to-skin contact within 24 hours after birth?	No...0<br>Yes.. 1	
4.5	Was there any liquid, paste or cream applied to the cord stump?	Yes...1<br><br>No ....0<br>Don't know 98	**4.7**
4.6	What was applied to the stump?<br><br>Circle all things mentioned,<br>but do NOT prompt with any suggestions.	Antiseptic..1<br>Oil...2<br>Vermillion ....3<br>Mud .... 4<br>Talcum powder...5<br>Turmeric ....6<br>Cloth ....7<br>Animal dung .... 8<br>**If others, write:**<br><br><br>Don't know...98	
4.7	How long after birth was your baby bathed for the first time?	Within the first 6 hours ....1<br>Between 7 and 24 hours .... 2<br>After 24 hours .... 3<br>Don't know............................... 98	
4.8	Did you ever start breastfeeding your baby?	No...0<br><br>Yes..1	**4.10**
4.9	How soon after birth did you first breastfeed?	During the first hour after delivery....................... 1<br>1-4 hours .... 2<br><br>More than 4 hours. ....3<br><br>Don't know.. 98	**5.1**<br><br><br>**5.1**
4.10	What reasons led you not to breastfeed your baby or not to breastfeed sooner after birth?	I had a C-section.. 1<br>Baby was taken away, s/he was not well .... 2<br>I was not feeling well .... 3<br>I don't think the first milk was good ....4<br>**If others, write:**<br><br><br>Don't know.. 98	

SECTION 5: NEWBORN CARE DURING FIRST MONTH

Interviewer: "Now, I would like to ask you some questions about the health of your child during the month after your most recent delivery."

<table>
<tr><th>No</th><th>Question</th><th>Codes</th><th>Skip</th></tr>
<tr><td>5.1</td><td>What signs or conditions would make a mother seek medical health services for her baby in their first month after birth?

Circle all signs mentioned,
but do NOT prompt with any suggestions</td><td>Poor sucking or feeding……………………………………………. 1
Fast or difficult breathing…………………………………………. 2
Feels cold or too hot……………………………………………….. 3
Difficult to wake/ lethargic/ unconscious …………………..4
Excessive crying ……………………………………………………. 5
Redness of skin around cord/foul smelling discharge.. 6
Blue skin colour ………………………………………………………7
Jaundice ………………………………………………………………. 8
No or delayed cry at birth ………………………………………..9
Cough…………………………………………………………………. 10
Diarrhoea……………………………………………………………… 11
Vomiting repeatedly……………………………………………… 12
Pustules/boils on skin …………………………………………… 13
Fits ……………………………………………………………………. 14
Redness in the eye/infection…………………………………..15
Congenital anomaly………………………………………………..16
Does not pass stool/urine ……………………………………….17
If others, write:

Don't know……………………………………………………………..98</td><td></td></tr>
<tr><td>5.2</td><td>If your baby is not well, who is the first and second from whom you seek help?

Mark in the table with X only one option for first and one for second</td><td>
<table>
<tr><th>Seek help…</th><th>1st</th><th>2nd</th></tr>
<tr><td>Doctor</td><td></td><td></td></tr>
<tr><td>Auxiliary Nurse Midwife</td><td></td><td></td></tr>
<tr><td>ASHA</td><td></td><td></td></tr>
<tr><td>Traditional Birth Attendant</td><td></td><td></td></tr>
<tr><td>Anganwadi worker</td><td></td><td></td></tr>
<tr><td>Pharmacist</td><td></td><td></td></tr>
<tr><td>Spiritual/religious leader</td><td></td><td></td></tr>
<tr><td>Family /friends (NON TRAINED)</td><td></td><td></td></tr>
<tr><td>Other, write</td><td></td><td></td></tr>
<tr><td>Don't know</td><td></td><td></td></tr>
</table>
</td><td></td></tr>
<tr><td>5.3</td><td>Did a nurse, doctor, the ASHA or any health worker check on your newborn's health during the following period of time?</td><td>
<table>
<tr><th></th><th colspan="3">Period</th></tr>
<tr><td>Options</td><td>First 3 days</td><td>4th day to 2 weeks</td><td>2-4 weeks</td></tr>
<tr><td>Yes</td><td>1</td><td>1</td><td>1</td></tr>
<tr><td>No</td><td>0</td><td>0</td><td>0</td></tr>
<tr><td>Don't know</td><td>98</td><td>98</td><td>98</td></tr>
<tr><td>Baby was not alive by then</td><td>2</td><td>2</td><td>2</td></tr>
<tr><td>Baby has not yet completed</td><td>3</td><td>3</td><td>3</td></tr>
</table>
</td><td>If coded '0' or '98' in ALL 3 columns OR

if coded '2' or '3' in "First 3 days":

Thanks and finish</td></tr>
</table>

5.4	Who checked on your newborn's health during this time?<br><br>Circle all persons mentioned, but do NOT prompt with any suggestions	Doctor .... 1<br>Auxiliary Nurse Midwife.......... .... 2<br>ASHA... .... 3<br>Traditional Birth Attendant ....4<br>Anganwadi worker ....5<br>**If others, write:**<br><br><br>Don't know 98	
5.5	During these visits which of the following information was given to you?<br><br>Read the options and circle :<br>0 for No<br>1 for Yes<br>98 for don't know	(see table below)	

	**No**	**Yes**	**Don't know**
Counselling on breast feeding	0	1	98
Keeping baby warm	0	1	98
Examination of the baby	0	1	98
Counselling on child's immunisation	0	1	98
Identifying danger signs in the newborn	0	1	98
Advice on cord stump care	0	1	98

THANK THE RESPONDENT!

TIME FINISHED

HOURS : MINUTES : [ ][ ] [ ][ ]

Interviewer ID _______Signature_________________________
